# Correlation of serum Klotho, fetuin-A, and MGP levels with coronary artery calcification in maintenance hemodialysis patients

**DOI:** 10.1016/j.clinsp.2024.100417

**Published:** 2024-07-31

**Authors:** Dan Wang, XiuLin Chu, JuHua Cao, YunHua Peng

**Affiliations:** a Department of East Hospital Nephrology, Yantaishan Hospital, Yantai City, Shandong Province, China; b Department of Nephrology, The People's Hospital of Xushui, Baoding City, Hebei Province, China; c Department of Outpatient, The General Hospital of Western Theater Command of Chinese people's liberation army, Chengdu City, Sichuan Province, China; d Department of Nephrology, Dafeng People's Hospital, Yancheng City, JiangSu Province, China

**Keywords:** Maintenance hemodialysis patients, Klotho, Fetuin-A, MGP, Coronary artery calcification

## Abstract

**Objective:**

This study was to investigate the role of serum Klotho, fetuin-A, and Matrix Gla Protein (MGP) in Coronary Artery Calcification (CAC) in patients with Maintenance Hemodialysis (MHD) and their predictive value for CAC.

**Methods:**

100 patients receiving MHD were selected. Serum Klotho, fetuin-A, and MGP levels were detected by ELISA. CAC scores were assessed by coronary CT scan. Multifactor analysis was used to evaluate the risk factors affecting CAC. The ability of serum Klotho, fetuin-A, and MGP levels to diagnose CAC was evaluated by receiver operating characteristic curves.

**Results:**

Serum Klotho, fetuin-A, and MGP were independent risk factors for CAC. Serum Klotho, fetuin-A, and MGP were valuable in the diagnosis of CAC in MHD patients.

**Conclusion:**

There is a close relationship between Klotho, fetuin-A, and MGP levels in MHD patients and CAC.

## Highlights

• Serum Klotho, fetuin-A, and MGP were independent risk factors for CAC.
• Serum Klotho, fetuin-A, and MGP were valuable in the diagnosis of CAC in MHD patients.
• There is a close relationship between Klotho, fetuin-A, and MGP levels in MHD patients and CAC.

## Introduction

Chronic Kidney Disease (CKD) is a growing health problem worldwide, affecting approximately 10%‒16% of the adult population worldwide.^1^ The prevalence of CKD in China has reached 10.8%,^2^ and the number of Maintenance Hemodialysis (MHD) patients is also gradually increasing. MHD patients are more likely to have vascular calcifications^3^ and Coronary Artery Calcification (CAC).^4^, ^5^, ^6^ In light of this, evaluating and preventing CAC is becoming increasingly important.

CAC is not a simple precipitation of calcium and phosphorus, but an active and constantly changing process,^7^ which is currently thought to be the deposition of an organized extracellular matrix caused by osteoblast-like cells. It is formed when Vascular Smooth Muscle Cells (VSMCs) undergo transdifferentiation as a result of factors such as hyperphosphatemia. VSMCs produce a matrix composed of collagen and non-collagen proteins. If pro-mineralization factors exceed inhibitory factors, the lesion will be mineralized,^8^ and the imbalance between pro-mineralization factors (hyperphosphatemia, inflammation) and the inhibitory factors (fetuin-A and others) ultimately leads to vascular calcification even in early CKD.

An aging suppressor gene named Klotho was first reported in 1997.^9^ As an FGF-23 co-receptor, Klotho is primarily expressed in the choroid plexus, distal convoluted tubules, and parathyroid glands.^10^^,^^11^ Various aging-related disorders have been linked to Klotho, including organ atrophy, premature atherosclerosis, calcified vascular and soft tissues, and cancer.^12^

The 62-kilodalton glycoprotein, feluin-A, is a cystatin-family member. As secreted from the liver, the 349-amino acid protein consists of a heavy and light chain that is joined and linked.^13^ Two cystatin domains exist at the N-terminus of the heavy chain, D1 and D2. Acidic amino acids in the D1 domain are believed to be responsible for the ability of fetuin to inhibit calcium and phosphorus precipitation.^13^ It is mainly due to fetuin-A that serum prevents calcium and phosphorus precipitation *in vitro*. Currently, fetuin-A is recognized as an active regulator of osteogenesis within the vessels. Fetuin-A binds to annexin II and I on cell surfaces in the presence of calcium.^14^ As a result of this binding, fetuin-A can enter the endosomes within VSMCs.^15^ Fetuin-A inhibits VMSC apoptosis intracellularly.^16^ Furthermore, it inhibits mineralization and suppresses calcium phosphate nucleation.^16^ In addition, fetuin-A inhibits bone morphogenetic protein-2, a key osteogenic protein that triggers the transdifferentiation of calcifying vascular cells.^13^

A vascular calcification inhibitor, Matrix Gla Protein (MGP), depends on vitamin K for its activation. A higher plasma level of desphospho-uncarboxylated MGP (indicative of poor vascular vitamin K status) is linked with an increased cardiovascular risk.^17^ MGP levels have been demonstrated to be high in Maintenance Hemodialysis (MHD) patients.^18^

However, the effects of serum Klotho, fetuin-A, and MGP levels on CAC and the predictive value of these biomarkers in MHD patients are unclear. This study investigated Klotho, fetuin-A, and MGP in CAC in MHD patients.

## Materials and methods

### Research objects

100 patients receiving MHD in Yantaishan Hospital were selected. MHD was performed with bicarbonate dialysate containing 1.5 mmoL/L Ca^2+^ on a polysulfone membrane dialyzer. Blood flow was 200‒250 mL/min, dialysate flow rate was 500 mL/min, and dialysate temperature was 37°C. The anticoagulant treatment was conventional heparin or low molecular-weight heparin. MHD was generally performed 4‒5h per time, 2‒3 times a week. An autologous arteriovenous fistula was established as vascular access. Patients with hypoproteinemia, active hectic diseases, hepatobiliary disease, severe infection, gastrointestinal bleeding, retinal bleeding, or other active bleeding conditions were excluded. Patients were divided into 3 groups according to CAC scores (0‒100, 100‒400, and > 400). Basic clinical data were recorded. This study was an observational clinical study following the Strengthening the Reporting of Observational Studies in Epidemiology (STROBE) guidelines.

### Biochemical analysis

Fasting venous blood was collected to detect hemoglobin, Albumin (Alb), blood calcium, and blood phosphorus levels. $\text{Total}\;\text{calcium}\;\left(\frac{\text{mg}}{\text{dL}}\right)=\;\text{total}\;\text{calcium}\;+\;0.8\;\times \;\left[4\;-\;\text{Alb}\;(\frac{\text{mg}}{\text{dL}})\right].$ The corrected total calcium is used to calculate the calcium-phosphorus product. Dialysis adequacy was evaluated by calculating the ratio of urea clearance to Volume (Kt/V).

### Serum Klotho, fetuin-A, and MGP levels

Fasting venous blood was centrifuged and cryopreserved for follow-up testing. Serum Klotho, fetuin-A and MGP levels were detected by ELISA, and then the correlation between serum Klotho, fetuin-A and MGP levels and CAC in MHD patients was analyzed. Specifically, serum Klotho levels were detected using an ELISA kit (IBL-International, Japan). Klotho in all samples exceeded the detection limit of 6 pg/mL. Intra-assay and inter-assay variations were 3.6% and 3.2% for the Klotho standard and 2.8% and 3.6% for the human sample, respectively. Serum fetuin-A levels were detected using an ELISA kit (Bio-Swamp, USA). The mean intra-assay and inter-assay variations were 5.6% and 6.2%, respectively. The reference range for healthy adults is 0.5‒1.0 g/L, with a minimum sensitivity of 2.5 ng/mL. Serum MGP levels were detected using an ELISA kit (Bio-Swamp). Intra-assay and inter-assay variations were 5.4% and 7.2%, respectively. The lower limit is 5 pg/mL.

### Isolation of peripheral blood mononuclear cells (PBMCs)

Venous blood (1.5 mL) was diluted with phosphate buffer at 1:1 and transferred into a centrifuge tube containing 3 mL Ficoll Paque (GE Healthcare, Uppsala, Sweden). PBMCs were collected after centrifugation at 400× g at room temperature for 20 min and rinsed twice in 10 mL PBS. PBMCs were re-suspended in a lysis buffer for protein extraction.

### Western blot

The protein expression levels of Klotho, fetuin-A and MGP in PBMCs of MHD patients were detected by Western blot, which in turn increased the reliability of the results of serum Klotho, fetuin-A and MGP levels. The cell lysate was prepared, and the protein concentration was detected by the BCA protein detection reagent. Protein (10‒30 μg) was electrophoresed on an 8% or 12% SDS-polyacrylamide gel, transferred to a PVDF membrane, and sealed at room temperature for 2h in 5% skim milk containing PBST. Subsequently, the membrane was incubated with the primary antibody at 4°C overnight and then re-detected with HRP-bound secondary antibody (1:3000) for 2h. Protein expression was finally shown using the ECL kit.

### CAC score

CT scans were performed using 64-slice spiral technology (Lightspeed VCT, USA). Continuous images were acquired by plain axial scan combined with retrospective electrocardio gram gating during diastole of the cardiac cycle from the carina to the apex. At 60%‒80% R-R interval, the images were obtained during a single breath-hold. Plain 3.0-mm images were reconstructed at a 40% R-R interval in the diastolic phase. CAC scores were calculated using SmartScore software (GE Healthcare, USA). Attenuation values over 130 hounsfield units were considered to be the cut-off point for calcification lesions. Areas greater than 0.5 mm^2^ were considered calcified. Left main, Left Anterior Descending (LAD), Left Circumflex (LCX), and right coronary artery were marked for calcification. LAD calcification refers to the calcification of diagonal branches, while LCX calcification refers to obtuse marginal branches. Total CAC scores were calculated automatically. CT scan (512×512 matrix; 25‒28 cm filed of view): peak voltage at 120 KV, tube current at 450 mA, collimator width of 1.5 mm, pitch of 0.24, scanning time for 5‒8s, gantry rotation time for 0.35s, slice thickness of 2.5 mm, and reconstruction interval of 2.5 mm.

### Carotid intima-media thickness (IMT) measurement

Common Carotid Artery (CCA) was examined from both longitudinal and transverse angles using a color Doppler ultrasound system (LOGIC 9, General Electric). During the left ventricular diastolic period, CCA images at 1.5‒2 cm below the carotid sinus were obtained. Measurements were repeated for 3 cardiac cycles on each side. There are two parallel bright lines in the posterior wall of CCA, and the echo intensity was relatively low. IMT was calculated based on the vertical distance between the two lines, which is the average of six carotid artery measurements. It was the same sonographer who performed each scan.

### Statistical analysis

Analyses were performed with SPSS 16.0 software. Two-sided tests were used for all statistical inferences; p < 0.05 was considered statistically significant. If the data fit the homogeneity of variance and the normal distribution, the comparison was done with an independent sample *t*-test; if not, the Wilcoxon rank sum test was used. One-way analysis of variance was used for comparison among groups, and the least significant difference method was for that between two means. Variance heterogeneity data were assessed by the Wilcoxon rank sum test. Continuous variables were analyzed by Principal Component Analysis (PCA), and categorical variables were analyzed by Multiple Correspondence Analysis (MCA) to build a random forest model to assess the importance of each variable. Then, multiple stepwise regression analysis was performed with CAC score as the dependent variable and gender (female = 0, male = 1), age, BMI, dialysis vintage, Kt/V, IMT, blood calcium level, blood phosphorus level, calcium-phosphorus product, iPTH, Klotho, fetuin-A, and MGP as independent variables. Partial regression coefficient and 95% CI were calculated. The Receiver Operating Characteristic (ROC) curve was plotted and Area Under the Curve (AUC) was analyzed.

## Results

### Patient baseline characteristics

Among the 100 patients, 46 (46.00%) were males and 54 (54.00%) were females. The mean age was 60.40±11.50 years, and the mean dialysis vintage was 6.79±2.85 years. The top 3 underlying diseases were chronic glomerulonephritis (26 cases, 26.00%), diabetes (18 cases, 18.00%), and hypertensive nephropathy (14 cases, 14.00%) (Table 1).

**Table 1:** General data and medical history of patients.

Parameters	MHD (n = 100)
Gender (male/female)	46/54 (46.00%/54.00%)
Age (years)	60.4 ± 11.5
Body mass index (kg/m2)	22.64 ± 0.57
Dialysis vintage (years)	6.79 ± 2.85
Chronic glomerulonephritis	26 (26.00%)
Diabetic nephropathy	18 (18.00%)
Hypertensive nephropathy	14 (14.00%)
Polycystic kidney	7 (7.00%)
Chronic interstitial nephritis	6 (6.00%)
Uric acid nephropathy	5 (5.00%)
Obstructive nephropathy	4 (4.00%)
Unknown etiology	20 (20.00%)

### CAC scores

Table 2 summarizes the characteristics of patients in the three defined groups of CAC scores 0‒100, 100‒400, and > 400. There were significant differences in BMI, dialysis vintage, Kt/V, IMT, blood phosphorus, calcium-phosphorus product, Klotho, fetuin-A, and MGP. With the increase of CAC score, MHD patients had increased dialysis vintage, higher BMI, IMT, blood phosphorus levels, calcium-phosphorus product, and lower Kt/V. The serum levels of Klotho, fetuin-A and MGP in MHD patients were measured by ELISA. With the increase in CAC score, serum MGP showed an increasing trend, and serum Klotho and fetuin-A showed a decreasing trend.

**Table 2:** Comparison of coronary artery calcification parameters in different groups.

	CAC score	CAC score	CAC score	
	0‒100	100‒400	> 400	
Parameters	(n = 20)	(n = 25)	(n = 55)	p-value
CAC score	48.32 ± 18.65	238.77 ± 85.03	1076.84 ± 340.59	<0.001
Gender (male/female)	11/9	10/15	25/30	0.600
Age (years)	59.85 ± 7.20	60.24 ± 6.56	60.67 ± 6.98	0.895
BMI (kg/m2)	21.45 ± 0.21	22.30 ± 0.16	23.23 ± 0.19	<0.001
Dialysis vintage (years)	4.20 ± 0.22	5.10 ± 0.27	8.50 ± 0.25	<0.001
Kt/V	1.28 ± 0.04	1.24 ± 0.02	1.20 ± 0.03	<0.001
IMT (mm)	0.76 ± 0.06	0.82 ± 0.04	0.95 ± 0.10	<0.001
Hb (g/L)	112.16 ± 8.48	109.94 ± 9.17	107.36 ± 9.85	0.131
Alb (g/L)	39.34 ± 3.57	38.51 ± 4.35	39.08 ± 4.74	0.803
Calcium (mmoL/L)	2.12 ± 0.10	2.09 ± 0.11	2.06 ± 0.12	0.119
Phosphorus (mmoL/L)	1.72 ± 0.34	1.95 ± 0.46	2.24 ± 0.25	<0.001
Calcium-phosphorus product (mmoL2/L2)	3.64 ± 0.45	4.08 ± 0.38	4.61 ± 0.22	<0.001
Klotho (pg/mL)	545.18 ± 64.25	526.34 ± 52.68	501.75 ± 46.93	0.005
Fetuin-A (mg/mL)	94.18 ± 6.15	87.49 ± 4.87	69.54 ± 3.16	<0.001
MGP (pg/mL)	170.07 ± 17.94	204.15 ± 13.16	309.51 ± 10.82	<0.001

### Klotho, fetuin-A, and MGP proteins in three defined groups of CAC scores

**Fig. 1:**
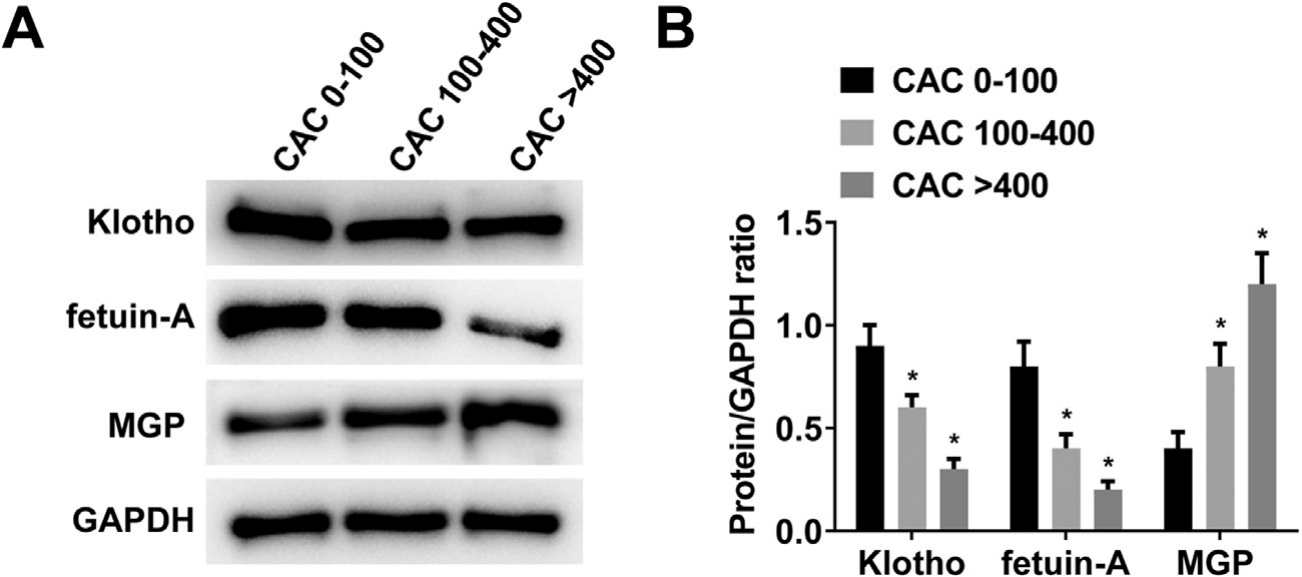
Klotho, fetuin-A, and MGP proteins in three defined groups of CAC scores. (A‒B) Protein expressions of Klotho, fetuin-A, and MGP in three defined groups of CAC scores were detected by Western blot.

Klotho, fetuin-A, and MGP protein expression levels in PBMCs of MHD patients in different CAC score groups were detected by Western blot. With the increase of CAC score, Klotho and fetuin-A proteins gradually decreased, while MGP proteins gradually increased (Fig. 1 A‒B).

### Analysis of influencing factors of CAC in MHD patients

Multiple stepwise regression analysis with CAC score as the dependent variable and gender (female = 0, male = 1), age, BMI, dialysis vintage, Kt/V, IMT, blood calcium level, blood phosphorus level, calcium-phosphorus product, iPTH, Klotho, fetuin-A, and MGP as independent variables showed that after controlling gender and age, dialysis vintage, blood phosphorus, serum Klotho, fetuin-A, and MGP were independent risk factors for CAC in MHD patients (Table 3).

**Table 3:** Multiple stepwise regression analysis of CAC scores in patients on MHD.

Variable	B	95%CI		p-value
		Lower	Upper	
Dialysis vintage (years)	6.4	4.16	19.35	0.024
Phosphorus (mmoL/L)	15.05	9.96	30.67	0.03
Klotho (pg/mL)	-0.56	-0.95	-0.31	0.027
Fetuin-A (mg/mL)	-9.69	-21.34	-5.05	0.039
MGP (pg/mL)	7.54	4.36	17.21	0.013

### Diagnostic value of serum Klotho, fetuin-A, and MGP for CAC in patients with MHD

**Fig. 2:**
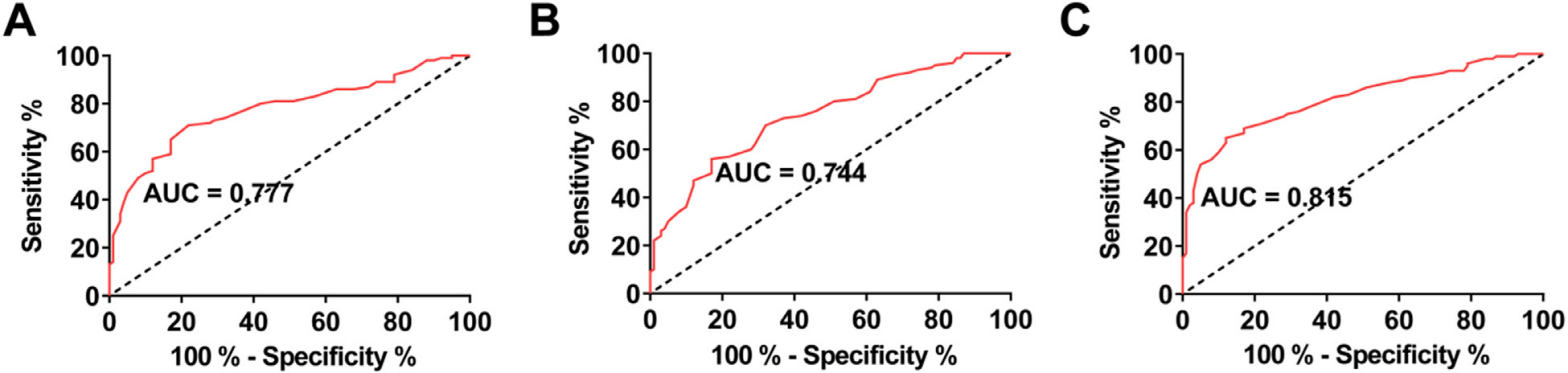
Diagnostic value of serum Klotho, fetuin-A, and MGP in CAC in MHD patients. (A‒C) ROC curve analysis of serum Klotho, fetuin-A, and MGP levels to predict CAC in MHD patients.

ROC curves were drawn to analyze the value of serum Klotho, fetuin-A, and MGP levels in predicting CAC in MHD patients. The AUC of serum Klotho, fetuin-A, and MGP was 0.777, 0.744, and 0.815, respectively (Fig. 2 A‒C).

## Discussion

Vascular calcification is common in patients with MHD. Data showed that 27% of patients receiving dialysis for 1 year found vascular calcification, and the incidence increased to 83% of patients receiving dialysis for more than 8 years.^19^^,^^20^ One hundred MHD patients with dialysis vintage of 6.79 ± 2.85 years were included in this study, of which 20 (20.00%), 25 (25.00%) and 55 (55.00%) had CAC scores of 0‒100, 100‒400 and > 400, respectively.

MHD patients often have renal phosphorus metabolism disorder, and intake of phosphorus-rich foods results in phosphorus retention and stimulate iPTH secretion. Calcium and phosphorus can be released from bones into the extracellular space due to excess iPTH, aggravating hyperphosphatemia, which is associated with vascular calcification. Hyperphosphatemia, calcium-phosphorus accumulation, and high calcium intake are associated with CAC.^3^ Vascular calcification can be alleviated by applying sevelamer hydrochloride actively to correct hyperphosphatemia.^21^ It has been indicated that inorganic phosphorus not only induces the transformation of VSMCs to osteoblast-like cells but stimulates osteoblast proliferation.^22^ It would seem that hyperphosphatemia may cause vascular walls to thicken, resulting in vascular calcification in patients with MHD. MHD and serum calcium and phosphorus levels are independent risk factors for CAC.^23^ For every 0.322 mmoL/L increase in blood phosphorus levels, the risk of developing CAC is comparable to that of 2.5 years of hemodialysis. According to a systematic review, dialysis duration and age are the most significant risk factors for CAC.^24^ In this study, the authors found that dialysis vintage and blood phosphorus levels were risk factors for the development of CAC.

The renal protective mechanism of Klotho against CKD has been reported previously. Klotho gene manipulation can improve renal function in mice with progressive kidney injury.^25^ Renal fibrosis is associated with Klotho deficiency, and Klotho treatment could prevent fibrotic kidney changes in mice.^26^ Klotho deletion results in increased renal inflammation through RelA (Serine)^536^ phosphorylation.^27^ In addition, Klotho can improve vascular calcification.^28^ In this study, serum fetuin-A levels were inversely associated with the severity of CAC. In addition, Klotho protein expression in PBMCs from MHD patients gradually decreased with increasing CAC score.

During arterial calcification, fetuin-A acts as a calcification inhibitor. It has been reported that serum fetuin-A level is negatively associated with CAC in 30 MHD patients.^29^ Moreover, serum fetuin-A levels are reduced in patients with calcification scores > 5, and serum fetuin-A < 0.29 g/L is associated with the risk of cardiovascular calcification progression.^30^ Moreover, the volume of coronary plaque in MHD patients is significantly larger.^31^ Similar to studies in MHD patients, serum fetuin-A i also significantly correlated with CAC score in non-dialysis ESRD patients.^32^ This study found that serum fetuin-A level was negatively correlated with the severity of CAC, which was consistent with the above studies. In addition, fetuin-A protein expression in PBMCs from MHD patients gradually decreased with increasing CAC score.

MGP can regulate intrachondral bone formation and vascular calcification and is associated with the downregulation of calcium deposition in the extracellular matrix of soft tissues. It has been suggested that elevated serum uncarboxylated MGP (ucMGP) is associated with aortic calcification.^33^ A negative correlation exists between ucMGP level and CAC in patients with CKD. UcMGP can be used as a proxy for the presence of vascular calcification. Another study^34^ has shown that baseline ucMGP levels are higher in MHD patients than normal. This study found that serum MGP level was positively correlated with the severity of CAC. In addition, MGP protein expression in PBMCs from MHD patients gradually increased with increasing CAC score.

In addition, the authors found that serum Klotho, fetuin-A, and MGP were biomarkers for the recognition of CAC in MHD patients with high sensitivity and specificity, indicating values for clinically predicting CAC.

## Conclusion

In conclusion, serum Klotho, fetuin-A, and MGP were closely associated with the severity of CAC in MHD patients and were expected to be promising diagnostic markers for CAC in MHD patients.

## Ethics approval

The present study was approved by the Ethics Committee of Yantaishan Hospital and written informed consent was provided by all patients prior to the study start. All procedures were performed in accordance with the ethical standards of the Institutional Review Board and The Declaration of Helsinki, and its later amendments or comparable ethical standards (201906GZ4).

## Data available

Data is available from the corresponding author on request.

## Authors' contributions

Dan Wang and XiuLin Chu the initiator of the study conducted the main experimental part, described the results, and wrote the article. JuHua Cao performed the statistical calculations, and participated in drafting of the manuscript. Dan Wang and YunHua Peng participated in collecting the research material. Lei Wang supervised the work.

## Conflicts of interest

The authors declare no conflicts of interest.
